# Phosphotungstic acid impregnated niobium coated superparamagnetic iron oxide nanoparticles as recyclable catalyst for selective isomerization of terpenes†

**DOI:** 10.1039/d1ra00012h

**Published:** 2021-04-15

**Authors:** Luccas Lossano Name, Sergio Hiroshi Toma, Helton Pereira Nogueira, Luis Humberto Avanzi, Rafael dos Santos Pereira, Luis Fernando Peffi Ferreira, Koiti Araki, Rodrigo Cella, Marcos Makoto Toyama

**Affiliations:** Department of Chemistry Engineering FEI University 3972B – Assunção – São Bernardo do Campo São Paulo CEP 09850-901 Brazil r.cella@fei.edu.br; Department of Fundamental Chemistry Institute of Chemistry, University of São Paulo, IQUSP Av Lineu Prestes, 748 – Cidade Universitária CEP 05508-000 São Paulo Brazil marcosmakotoyama@gmail; Department of Physics FEI University 3972B – Assunção – São Bernardo do Campo São Paulo CEP 09850-901 Brazil; Department of Physics, Universidade Federal do ABC, Centro de Ciências Naturais e Humanas Avenida dos Estados, 5001 – Bloco A – Torre 3 – Lab. L704-3 – 09210580 – Bangu – Santo André SP Brazil

## Abstract

Conversion efficiency as high as 80–100% and 50% selectivity for camphene and limonene was achieved with low production of polymeric byproducts (18–28%), easy recovery with a magnet and reuse for up to five cycles maintaining similar activity and distribution of products, using a new magnetically recyclable catalyst based on niobium oxide coated on superparamagnetic iron oxide nanoparticles (SPION) impregnated with phosphotungstic acid (HPW). The catalyst was demonstrated to be effective in the selective conversion of alpha and beta-pinenes into valuable terpenes, under ultrasonic probe activation and with toluene as solvent. A unique synergic effect between the components generating more active and selective catalytic sites was demonstrated, indicating that the SPION covered with 30 wt% of Nb_2_O_5_ gives the best performance when impregnated with HPW as co-catalyst. The materials were fully characterized by XRD, EDX, XPS, TEM, BET, VSM and FTIR.

## Abstract 2

Conversion efficiency as high as 80–100% and 50% selectivity for camphene and limonene was achieved with low production of polymeric byproducts (18–28%), using a new magnetically recyclable catalyst – SPION-Nb30@HPW.

## Graphical abstract

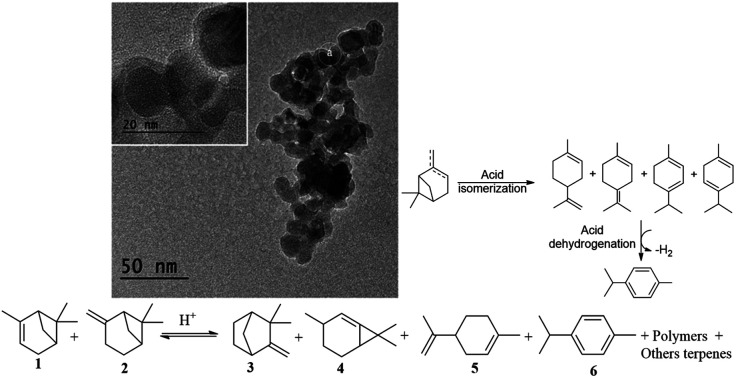

## Introduction

Bio-based products and biofuels are commonly seen as sustainable alternatives to chemicals derived from petroleum and related fossil fuels. Accordingly, products obtained from microorganisms and plants such as lignocellulose biomass, lipids and carbohydrates have proven to be a recent and strong promise for a more sustainable society.^1–3^ Terpenes, such as turpentine oil (mixture of α- and β-pinene), mainly extracted from coniferous trees, is an example of biomass that has caught the attention of many researchers in the past 25 years.

**Scheme 1:**
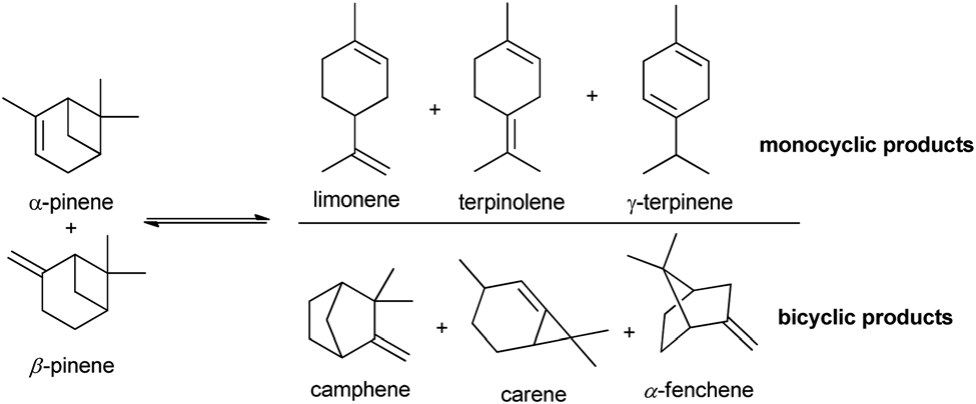
Main isomerization products of α- and β-pinene.

The isomerization of pinenes is an acid-catalysed reaction and can produce valuable terpenes such as camphene and limonene, as shown in Scheme 1, two important intermediates/ingredients in the cosmetics, food and pharmaceutical industries.^4–10^ Camphene is used for fabrication of isoborneol, isobornyl acetate and camphor, having more commercial applications than any other terpene. Industrial production of camphene is carried out in a closed system under relatively high temperatures (150–170 °C) and moderate yields ranging from 35% to 45%, using an acidic TiO_2_ catalyst.^11^ Thus, the development of more efficient and selective catalysts for conversion of pinenes to terpenes is relevant.

Nanostructuration provides interesting opportunities for development of materials for desired purposes such as catalysts, given the possibility of tailoring and optimizing their properties. In fact, nanocatalysts have much more intrincated structures than bulk materials, and present much higher surface area for a same volume, awarding them unique properties, especially higher density of more active and selective catalytic sites.^12^

Such materials can be prepared by incorporating catalytic active sites in suitable supporting materials. In this way different nanocatalysts can be generated using silica, carbon, alumina, and polymers as support since the activity is dependent on the interaction of the active sites with the supporting material.^13–16^ Among them, magnetite (Fe_3_O_4_) and maghemite (γ-Fe_2_O_3_)^17,18^ nanoparticles are been considered promising candidates since they are inexpensive, chemically stable, easy to prepare, and may be recovered by using an external magnetic field. In addition, magnetic nanoparticles are known for applications in several areas, such as drug delivery systems,^19–21^ targeted gene therapy,^22–24^ magnetic resonance imaging (MRI),^25^ sensors,^26^ separation and environmental remediation processes,^27^ nanomedicine^28–30^ and catalysis,^31–33^ additive of synthetic base oil,^34^ analytical chemistry using the SERS effect,^35,36^ among others.

Niobium-based materials incorporated in zeolites and mesoporous molecular sieves have been used to enhance several organic reactions.^37–39^ Accordingly, the binding of niobium oxide (Nb_2_O_5_) on the surface of superparamagnetic iron oxide nanoparticles (SPIONs) can generate new junctions and sites with enhanced catalytic activity upon synergic interactions between both metal oxides.^40^ In fact, sites coupling Lewis and Bronsted acidity with redox properties can be generated thus offering new mechanistic pathways for different reactions, but especially for α- and β-pinene isomerization reactions. In addition, the superparamagnetic property of the iron oxide nanoparticles can be exploited for easy magnetic recycling and reuse of the catalyst.

Finally, the occurrence of chemical reactions is strongly dependent on energy, and how it is provided. For example, it is well stablished that microwave is a very convenient way to increase very rapidly the local temperature decreasing the reaction time. Another alternative strategy to conventional heating is the use of ultrasonic probes which produce very high temperatures at the surface due to cavitation processes leading to the production, growth and fast collapse of bubbles. They are responsible for hot spots which can reach temperatures above 5000 K due to their very fast energy exchange.^41^ Thus, cavitation has been demonstrated to enhance the reaction kinetics, the selectivity and the yield of several organic reactions.^42^

Herein, the preparation of a new nanocatalyst based on synergic interactions of niobium oxide coated SPION impregnated with HPW co-catalyst, and its successful application for isomerization of turpentine oil (mixture of pinenes) to valuable terpenes, using ultrasound probe as energy source, is described.

## Results and discussion

### Characterization

**Fig. 1:**
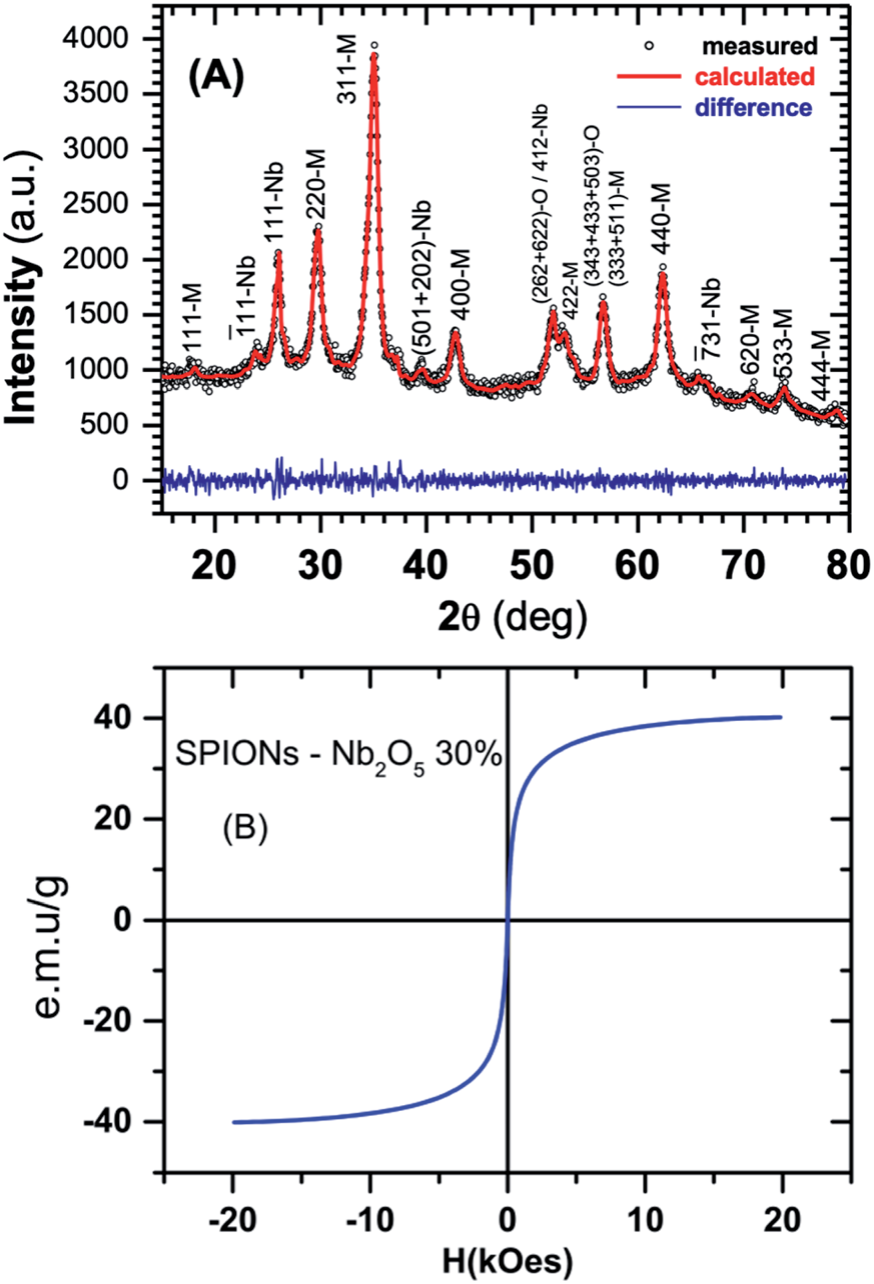
(A) Measured and calculated X-ray diffraction. The blue line in the bottom shows the fitting residual. The indices M, Nb and O that follows the reflection planes stands for Magnetite, Nb_2_O_5_ and NbO_2_ phases, respectively. (B) VSM of SPION-Nb30 pro-catalyst (complete cycle from −20 to 20 kOe and back to −20 kOe).

X-ray measurements was performed in a D2 Phaser (Brucker) diffractometer in Bragg–Brentano geometry (*θ*–2*θ* scan) and the diffraction pattern is depicted in Fig. 1A. Rietveld analysis shows that SPION-Nb30 (see Experimental section for detailed description) has 69.8 wt% of cubic magnetite, 29.1(7) wt% of monoclinic Nb_2_O_5_ (zeta-Nb_2_O_5_) and a small amount (1.1 wt%) of tetragonal NbO_2_. Crystallographic lattice parameters for both phases and the fitting results are shown in Table 1.

Crystallographic information for SPION-Nb30 pro-catalyst obtained from Rietveld refinements

**Table:** 

	Magnetite (Fe_3_O_4_)	Zeta-Nb_2_O_5_	NbO_2_
*a* (Å)	8.3892 (81)	13.063 (10)	13.71
*b* (Å)	—	5.7822 (62)	13.71
*c* (Å)	—	4.8034 (51)	5.985
*γ* (deg)	—	103.293 (66)	—
% wt	69.8	29.1 (7)	1.1(1)
Crystallite size (Å)	142.9 (2.9)	187 (14)	—
Crystal lattice symmetry	Cubic	Monoclinic	Tetragonal

**Table:** 

Goodness of fit (gof)		
*R*_wp_ = 2.97%	*R*_exp_ = 2.37%	*σ* = 1.25

The analysis was performed with MAUD software^43–45^ and compared with the diffractograms of single phase materials in Crystallography Open Database.^46^ Magnetic properties were investigated at room temperature using a vibrating sample magnetometer (VSM).

Magnetization curves clearly indicated the superparamagnetic behaviour of SPION-Nb30, characterized by absence, or very low hysteresis, and a saturation magnetization around 40 emu g^−1^ (Fig. 1B), contrasting with the original magnetization of 80 emu g^−1^ of pure SPION. The reduction on saturation magnetization was attributed to the diamagnetic contribution of the niobium oxide (Nb_2_O_5_) coating, which diminish the magnetic contribution of SPION itself. Some reaction should also be taking place, presumably on the surface of nanoparticles, since the magnetization is lower than expected by the relative contents of magnetite and Nb_2_O_5_ (∼56 emu g^−1^), as detailed below. The final magnetization however is large enough to induce efficient magnetic precipitation/separation of the catalyst.^27,33^

**Fig. 2:**
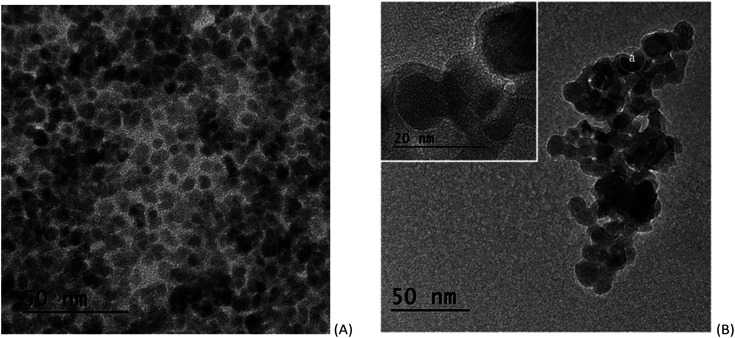
TEM images of SPION (A) and SPION-Nb30 pro-catalyst (B).

**Fig. 3:**
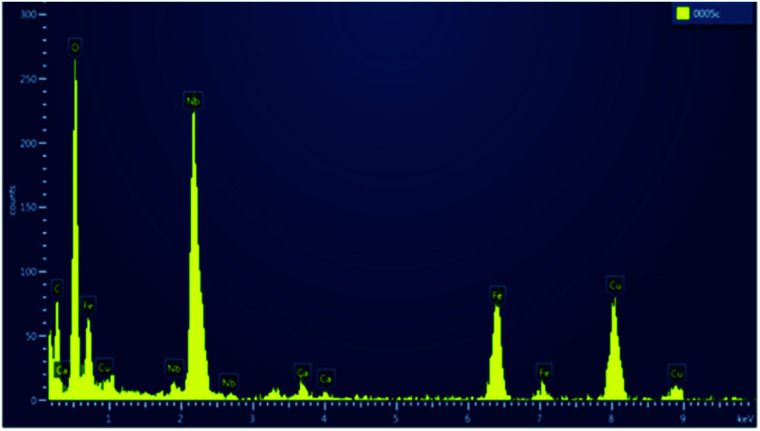
Selected area EDS spectrum of SPION-Nb30 pro-catalyst, shown in Fig. 2B.

TEM images of the SPION precursor and the corresponding SPION-Nb30 are shown in Fig. 2. The precursor nanoparticles are characterized by quasi-spherical shape, with an average diameter of 6 nm (Fig. 2A). Deposition of niobium oxide on SPION results in an increase of the average diameter to 13 nm, due to the formation of a less dense shell of Nb_2_O_5_ (Fig. 2B). This was confirmed by selected area EDS analysis that revealed the elemental composition and the presence of niobium oxide on the SPION core (Fig. 3).

**Fig. 4:**
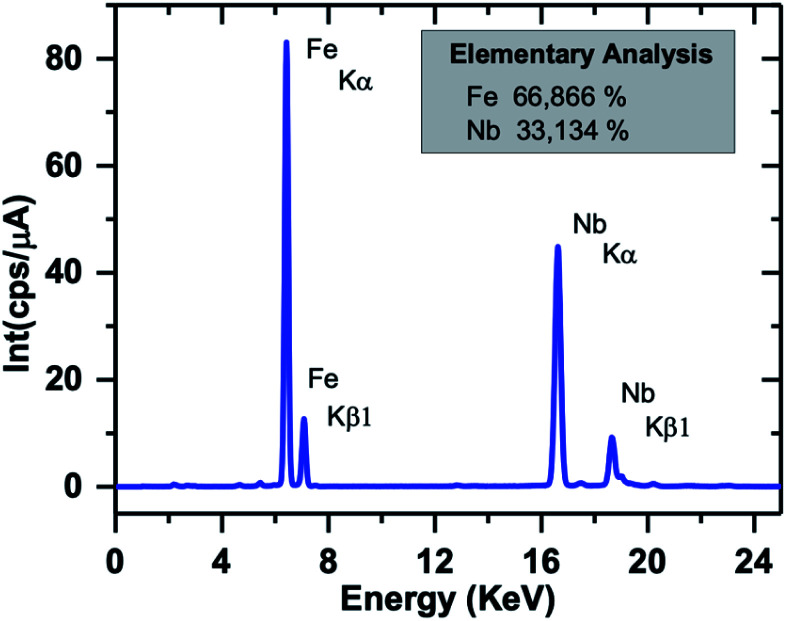
EDX spectrum of SPION-Nb30 sample showing the Fe and Nb fluorescence peaks.

The EDX spectrum of a typical SPION-Nb30 sample is shown in Fig. 4 indicating that it is constituted by 66% of Fe and 33% of Nb, in good agreement with the relative proportion of 69.8 wt% of cubic magnetite and 29.1 wt% of monoclinic Nb_2_O_5_ (zeta-Nb_2_O_5_) phases determined by XRD analysis.

Detailed synthetic procedure of SPION-Nb30 is described in the experimental section. It is important to comment that batches of SPION coated with 20, 30 and 50% of Nb_2_O_5_ were prepared and their respective catalytic activity for pinene isomerization evaluated. The best performance was achieved for the sample containing 30 wt% of niobium oxide, albeit the lower surface area, as shown in Table 2, where the parameters determined from the adsorption isotherm of SPION-Nb30 by BET analysis were collected. This is a clear indication that new catalytic active sites were formed at the junctions of the two oxides, since the material prepared with 50% of Nb_2_O_5_ of niobium oxide exhibited much lower catalytic activity.

**Table:** BET data (surface area, pore volume and pore size) for SPION-Nb30 and SPION-Nb50 determined by analysis of the N_2_ adsorption isotherm curves

Material	SPION-Nb30	SPION-Nb 50
Surface area (m^2^ g^−1^)	67.46	71.00
Pore volume (cm^3^ g^−1^)	0.2199	0.3307
Pore size (nm)	13.04	18.63

### Isomerization of turpentine

The catalytic activity of SPION-Nb30 for pinene isomerization reaction was studied aiming the production of camphene and limonene with the highest as possible selectivity. The set of initial parameters used for the isomerization reaction were temperature 80 °C, 5 wt% of catalyst in turpentine content, and reaction time of 8 h (Table 3 – entry 1). Boiling pearls were added to avoid liquid projection once magnetic stirring should be avoided in order to ensure the best dispersion of the superparamagnetic catalyst, but no conversion was observed under such a mild condition. In the second experiment, ultrasonic waves were employed as energy source for isomerization,^47–49^ however no conversion was observed either in this attempt (entry 2, Table 3).

**Table:** Screening the best condition for isomerization of turpentine oil^a^

No.	Catalytic system	Solvent	Conversion^b^ (%)	Selectivity^b^ (%)					
				3	4	5	6	Polymers	Others terpenes
1	SPION-Nb 30^c^	—	0	—	—	—	—	—	—
2	SPION-Nb 30	—	0	—	—	—	—	—	—
3	SPION-Nb 30 + HPW^c^	—	98	13	7	11	16	54	9
4	SPION-Nb 30 + HPW^a^	—	98	13	8	10	15	42	12
5	HPW	—	100	5	11	6	9	52	17
6	SPION-Nb 30 + HPW	Hexane	99	17	6	13	4	47	13
7	SPION-Nb 30 + HPW	Toluene	66	25	2	31	9	28	5
8	SPION-Nb 30	Toluene	0	—	—	—	—	—	—
9	HPW	Toluene	99	13	—	23	4	44	16

a Conditions: turpentine (2 g), catalyst (5wt%), solvent (2 mL), 30 minutes of sonication.

b Average of minimum two runs and yields calculated by GC.

c Oil bath at temperature of 80 °C for 8 h.

Accordingly, the SPION-Nb30 was activated with H_3_PW_12_O_40_ (HPW), an heteropolyacid known to impart higher activity than conventional acidic catalysts such as mineral acids, zeolites and ion-exchange resins. HPW has a Keggin type structure and possess interesting properties such as good thermal stability, high acidity and high oxidizing character.^50^ However, is less corrosive than mineral acids and do not promote secondary reactions, such as sulfonation and chlorination,^51,52^ and its use as catalyst or co-catalyst in pinene isomerization^53^ or hydration^54^ is well documented in the literature.

**Scheme 2:**
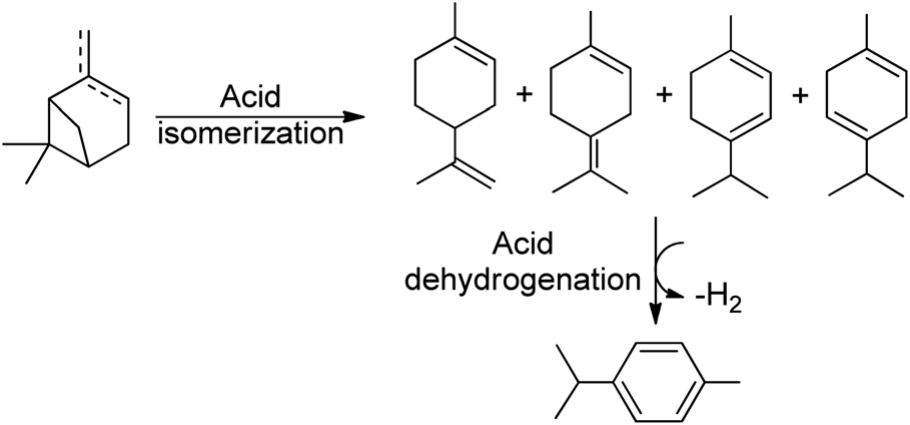
Scheme showing the dehydroisomerization of terpenes to *p*-cymene.

Thus, the SPION-Nb30 was treated with HPW generating a catalytic system with Lewis and Bronsted acidic sites, as well as redox properties, offering new mechanistic pathways for pinene isomerization. This catalytic system was tested under conventional heating (80 °C) affording 100% conversion of turpentine and 13% selectivity for camphene and 16% for *p*-cymene (entry 3, Table 3). However, the conversion to *p*-cymene is a more complex reaction and depends on an isomerization followed by a dehydrogenation process (Scheme 2).^55^ Carene 4, another bicyclic terpene, was afforded in significant amount also (selectivity 7%). Nevertheless, more than 50% of the products were undesirable polymers.

Accordingly, isomerization reactions were carried out using HPW as catalyst under the same experimental conditions in order to evaluate the role of SPION-Nb30. Analogously to the previous case, a high conversion was afforded however again with low selectivity. Thus, the use of ultrasonic tip as energy source was evaluated in the next attempt (entry 4, Table 3) providing very interesting results, since the amount of polymeric byproducts was reduced in about 12% while preserving the overall conversion yield.

As the reaction under ultrasound irradiation is much faster and finished in only 30 minutes in contrast with 8–12 hours under conventional heating, we decided to adopt the high intensity ultrasound (HIU) tip as energy source for pinene isomerization reaction. A reaction was carried out using just HPW as catalyst to demonstrate the role of SPION-Nb30 for the catalytic activity, and the result was included in Table 3 (entry 5). This reaction gave a much lower selectivity for camphene (only 5%) but larger amounts of polymers despite the excellent conversion of 100%, suggesting possible synergic effects between SPION-Nb30 and HPW.

In an attempt to minimize the formation of terpene polymers and improve the camphene yield, the control of the local reaction environment using organic solvents was considered relevant. Additionally, the effect induced by dilution and the possibility of tuning the environment around all species in the reaction medium making it more aliphatic or aromatic would be relevant for the stabilization of key activated complex species for the isomerization reaction. For such a purpose, hexane and toluene were tested (entries 6 and 7, respectively, Table 3), as suggested by Flores-Holguín and coworkers.^56^ The reaction using SPION-Nb30@HPW system and *n*-hexane as solvent showed an excellent conversion of 100% after 30 minutes of sonication, but the camphene selectivity was just a little higher than in the no solvent condition, and the amount of polymeric material was still as high as 50%.

The substitution of hexane by toluene as solvent in the above described reaction afforded a much lower conversion (66%) but the selectivity for camphene was boosted to 25%, as well as for limonene (31%), another very relevant product. Surprisingly, the use of toluene as solvent also have the additional advantage of decreasing the amount of polymers to about half when compared to the previous trials. In fact, only 25% of polymeric material was formed after 30 minutes of sonication indicating that toluene is a key solvent to reduce the amounts of undesirable polymers and enhance the production of valuable terpenes.

Isomerization reactions using only SPION-Nb30, or only HPW, as catalysts and toluene as solvent were also carried out (entries 8 and 9, Table 3) but showed to be as ineffective as in the solventless condition using only SPION-Nb30 as catalyst, resulting in no conversion at all. In contrast, HPW alone as catalyst in toluene lead to 99% conversion but the camphene selectivity was low (13%) and the amounts of polymeric products increased to 44%.

### Stability and regeneration of the catalyst

The reusability of the SPION-Nb30@HPW catalyst system was also studied. After the reaction, the catalyst was magnetically precipitated (recovered) in the flask bottom, washed with toluene several times, dried in an oven at 100 °C for 4 hours, and then reused in the reaction after impregnation with HPW. The reaction data are listed in Table 4. The catalytic activity is quite interesting since the conversion efficiency increased to 97% in the second cycle, but dropped to about 80% in the third and fourth reuse cycles while maintaining the selectivity for the desired products more or less constant in about 50%. These results are consistent with the hypothesis that HPW behaves as inorganic strong acid like H_3_PO_4_ generating new catalytic active sites on the surface of SPION-Nb30 while incorporating Bronsted acidic catalytic properties. The lower conversion efficiency of 66% in the first cycle (Table 4, entry 1) indicates a lower impregnation and/or formation of more active catalytic sites responsible for the higher conversion yields in the subsequent batches, while keeping the quantity of polymeric by-products low and preserving the selectivity for camphene and limonene (entries 2, 3 and 4). However, a tendency of lowering the yield of camphene accompanied by formation of larger amounts of polymers is apparent in the fifth reuse cycle. Such a lowering in conversion efficiency as a function of reuse cycle can possibly be assigned to poisoning and other undesirable changes in the surface chemistry.

**Table:** Magnetic recovery, reusability, and W leaching rate during isomerization of pinenes using SPION-Nb30@HPW as catalyst

Run	Conversion (%)	Selectivity (%)				Leaching (mg kg^−1^)
		3	5	Polymers	Others terpenes	
1	66	25	31	28	16	114
2	97	24	17	18	41	59
3	79	25	29	24	22	88
4	78	23	24	25	28	222
5	79	18	25	33	34	139

**Fig. 5:**
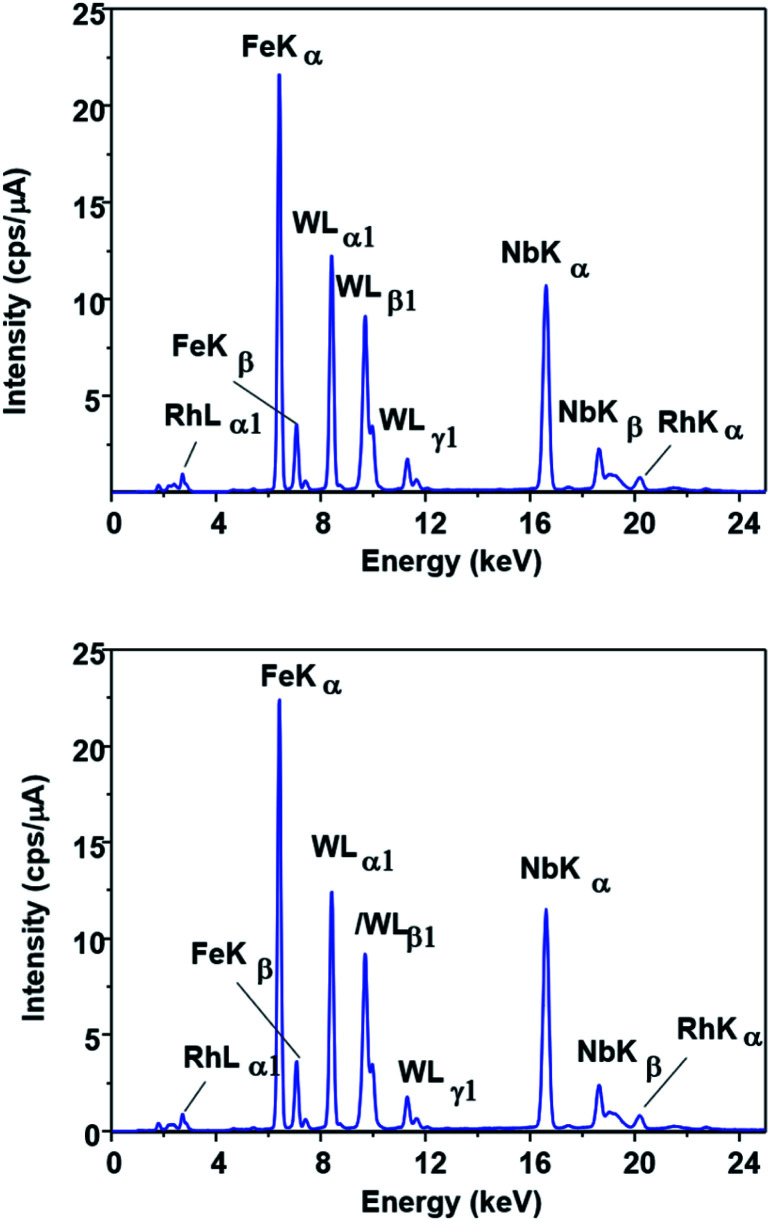
EDX spectra of (A) SPION-Nb30@HPW-R1 and (B) SPION-Nb30@HPW-R5 samples showing the similarity of the Fe, Nb and W fluorescence spectral patterns.

**Fig. 6:**
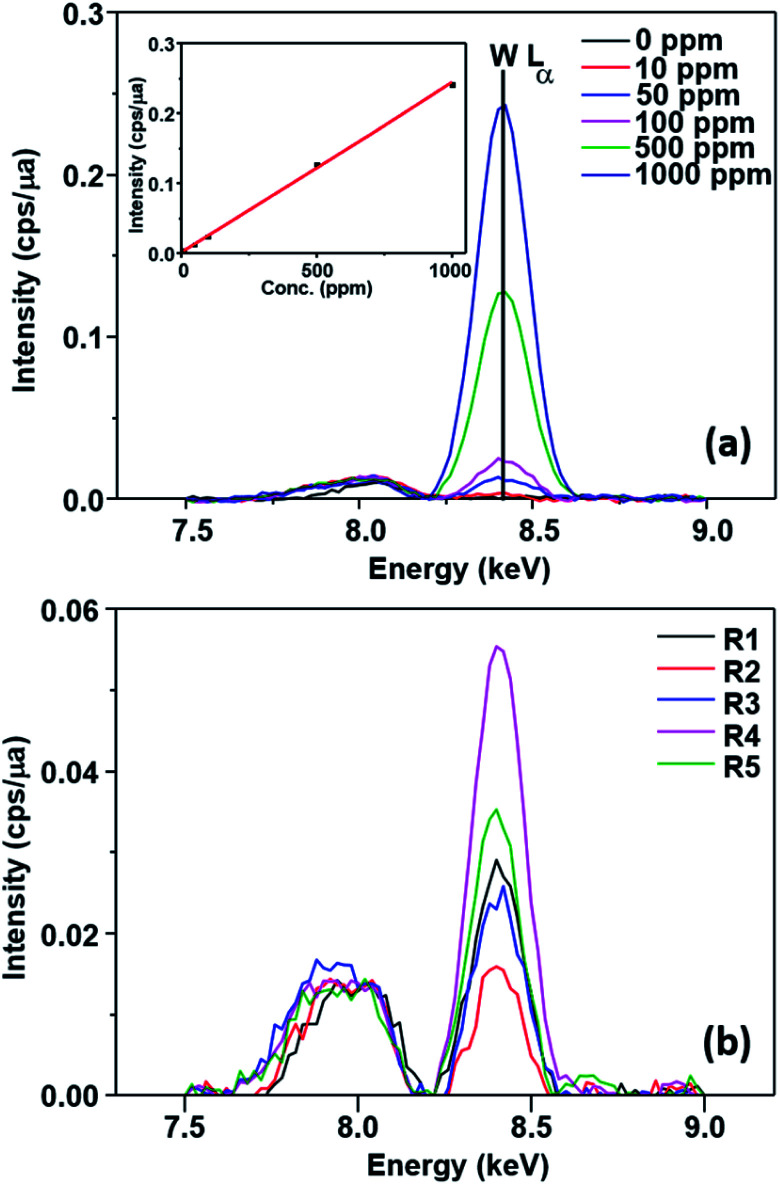
(A) Tungsten EDX spectrum of HPW solutions in the 0 to 1000 ppm concentration range and respective calibration curve (inset); and (B) EDX spectra of SPION-Nb30@HPW after the first (R1) up to the fifth reuse run (R5) showing the W fluorescence peaks of the reaction medium after magnetic removal of the catalyst, demonstrating the very small leaching of HPW from the catalyst.

This possibility was evaluated by FTIR, EDX and XPS measurements. No change could be observed in the EDX spectrum of the catalyst (Fig. 5) after the first run (SPION-Nb30-1R) and after the fifth run (SPION-Nb30-5R). This result indicates no significant loss/leaching of any component of the catalyst during the isomerization reaction as well as magnetic recovery and transfer and washing steps. However, a small leaching of HPW, the most soluble component, was revealed by monitoring the EDX W L_α_ signal (Fig. 6) in the supernatant of the reaction mixture, giving a measure of the amount leached per run cycle. In fact, 59 to 222 mg kg^−1^ of W was found in the reaction medium of each reuse cycle after magnetic precipitation and removal of the catalyst, as expected for a very small amount of weakly anchored HPW on SPION-Nb30 particles. The amount of 114 mg kg^−1^ determined in the first run decreased to 59 and 88 mg kg^−1^ of W respectively in the second and third reuse cycles, but increased to 222 and 139 mg kg^−1^ of W in the fourth and fifth run, as shown in Fig. 6B. Accordingly, the yield of compounds 3, 5, of other terpenes and polymers remained nearly constant from run 1 to 5, except in the second run where a significant decrease of 5 and polymeric products was compensated by an increase of the yield of other terpene derivatives (Table 4). A tendency of increasing polymer formation as a function of the reuse cycle was also evidenced suggesting some change in the catalyst.

**Fig. 7:**
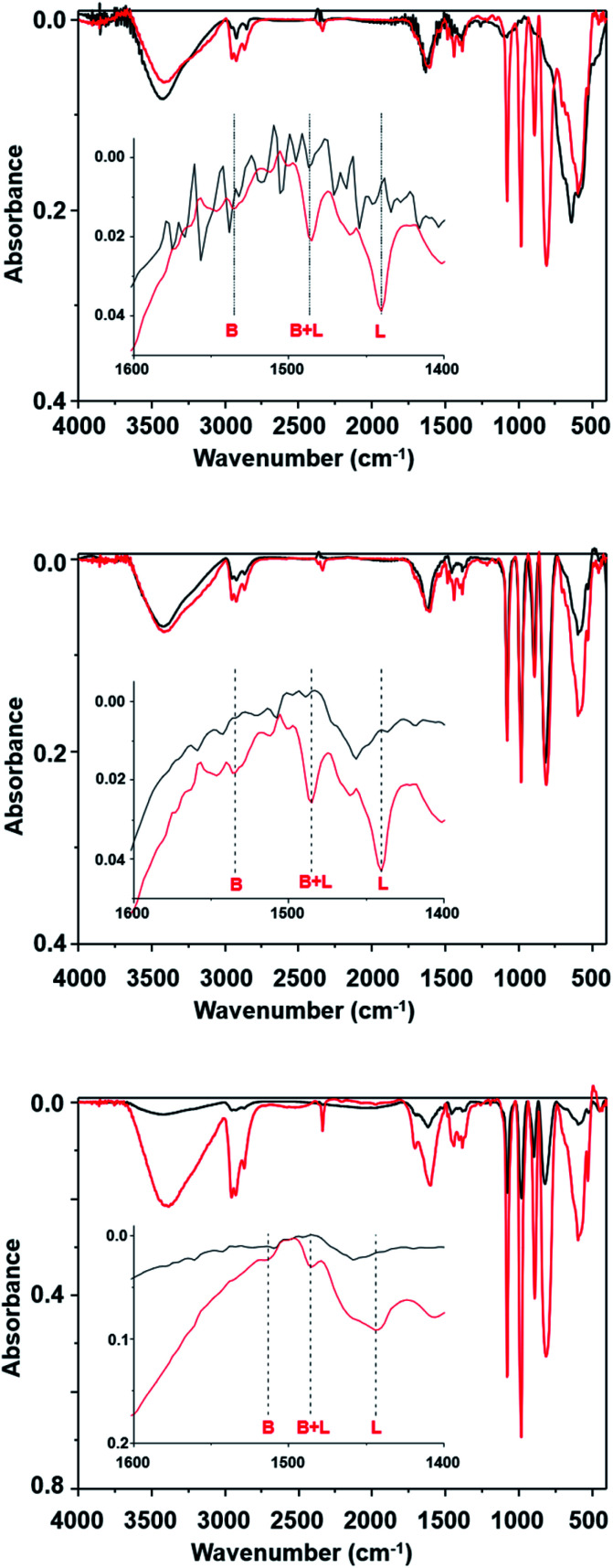
FT-IR spectra of (A) SPION-NB30, (B) SPION-Nb30@HPW-R1, and (C) SPION-Nb30@HPW-R5 magnetically recovered after the first and fifth catalytic cycle, before and after saturation with pyridine, showing the presence of Bronsted and Lewis acid sites, as well as the presence of adsorbed organic molecules.

The changes observed in the fifth run could be related to the suppression of the acidic catalytic active sites. This hypothesis was verified by determining the presence of Bronsted and Lewis acid sites based on the pyridine adsorption assay, as monitored by FTIR spectroscopy analysis. The FTIR of SPION-Nb30 (Fig. 7A) show a typical spectral profile of metal oxides with some low intensity peaks at 3000 cm^−1^ indicating the presence of some adsorbed organic molecules. The broad band at 3500 cm^−1^ as well as the should around 1700 cm^−1^ can be assigned to humidity. Interestingly, the adsorption of pyridine lead to the appearance of intense and sharp peaks in the 800 to 1250 cm^−1^ range, as well as of three peaks in the 1400 to 1600 cm^−1^ range used to identify the presence of Bronsted (1535 cm^−1^) and Lewis (1440 cm^−1^) acid sites, and the mixture of both (1485 cm^−1^). As indicated in the figure, the SPION-Nb30 presents almost only Lewis acid sites. Interesting enough, the FTIR spectrum of the SPION-Nb30@HPW-R1 after the first catalytic cycle (run 1, Fig. 7B) in the absence of and saturated with pyridine exhibited a similar spectral profile in the 800 to 1250 cm^−1^ range, that is also similar to the pyridine saturated SPION-Nb30 spectrum, indicating the presence of some strongly adsorbed molecules and/or a fast ligand exchange. The material recovered after the fifth catalytic cycle (run 5, Fig. 7C) exhibited a similar spectral pattern in the 800 to 1250 cm^−1^ range but now with much lower intensities and significant decrease of the concentration of acidic sites, especially Bronsted sites. These facts suggest changes in the surface chemistry and a decrease in the amount of previously adsorbed molecules. This feature can be related with a lower bonding strength of molecular species into associated with a lower density of acidic catalytic sites, that may explain the change in the catalyst performance (run 5 in Table 4).

**Fig. 8:**
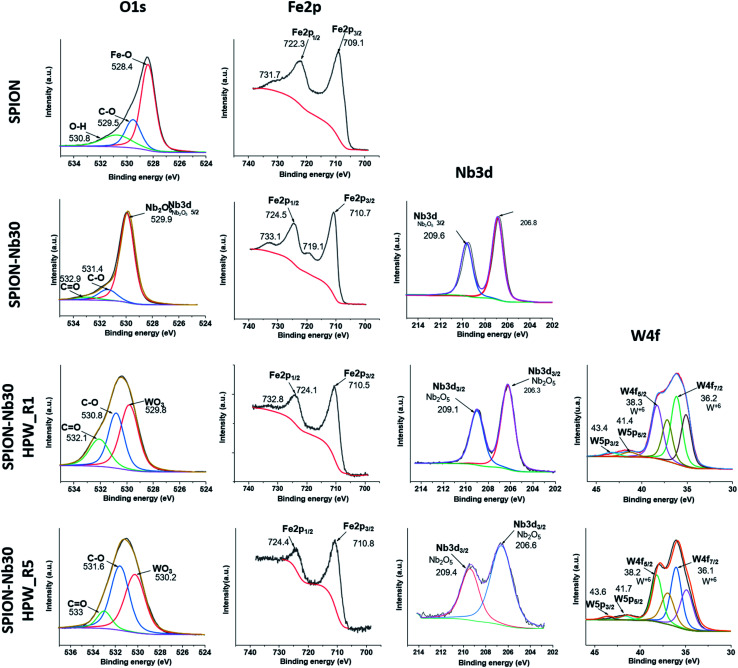
High resolution XPS spectra of SPION, SPION-NB30, SPION-Nb30@HPW-R1 (run 1), and SPION-Nb30@HPW-R5 (run 5) magnetically recovered from the reaction mixture after the first and fifth catalytic cycle.

Additional information was pursued by X-ray fluorescence (XPS) analysis of the catalyst after the first (SPION-Nb30@HPW-R1) and the fifth run (SPION-Nb30@HPW-R5) in comparison with the pure SPION and SPION-Nb30 pro-catalyst, as shown in Fig. 8 (high resolution spectra) and the corresponding survey spectra (Fig. S9, ESI†).

The SPION spectrum in the first line indicates de presence of iron oxide as magnetite and some amount of organic protecting layer as indicated by the presence in the C_1s_ region of a strong C–C carbon signal as well as C–O–C and carboxylic groups in the 278 to 292 eV region (Fig. S8, ESI†), that are confirmed by the O_1s_ signals (524 to 537 eV), as well as Fe 2p^1/2^ and Fe2p^3/2^ doublet (doubled due to the presence of Fe^2+^ and Fe^3+^ ions) characteristic of magnetite in the 700 to 740 eV range. As expected, the SPION-Nb30 exhibited similar signals in the C_1s_ O_1s_ and Fe 2p regions as well as a typical Nb 3d^3/2^ and doublet 3d^5/2^ signal in the 202 to 213 eV range. This spectral pattern is completely preserved in the SPION-Nb30@HPW-R1 except for the appearance of the signals assigned to W 4f^5/2^ and W 4f^7/2^ and satellites, as well as the W 5p^3/2^ and W 5p^5/2^ and satellites in the 32 to 46 eV range, and P 2P signal at 134 eV (Fig S8, ESI†), as expected for the impregnation of HPW (reported in ESI, Fig. SX†). The presence of HPW is also confirmed by the appearance of a new O 1s peak at 530 eV assigned to O-atom in WO_3_. Interestingly enough, the Nb 3d signal was significantly broadened in the SPION-Nb30@HPW-R5 indicating a change in its chemical environment after five recovery and reuse process, but especially it is possible to see the disappearance of the doublet corresponding to the Fe^2+^ 2p indicating the oxidation of magnetite to maghemite. In short, the changes in the oxidation state of iron, in the chemical environment of Nb and the decrease in concentration of acidic sites, especially of Bronsted acid sites, may be responsible for the increase in polymer yield as a function of the number of reuse cycles.

## Conclusion

The pinene isomerization reaction was chosen as model to study the catalytic activity and reusability of SPION-Nb30 impregnated with HPW. The superparamagnetic character conferred by SPION is fundamental since avoid self-agglomeration while incorporating the possibility of magnetic precipitation and recovery. This strategy is more convenient and faster than using filtration and conventional precipitation techniques for separation of the catalyst from the reaction medium, in addition to save energy and time. A reusable and efficient catalyst for isomerization of turpentine oil (80 to 100% conversion of pinenes) to valuable terpenes such as camphene and limonene (∼50%) was achieved by treatment of SPION-Nb30 with HPW. This combination generates new Lewis and Bronsted acidic active sites and/or a synergic effect, as induced by the use of toluene as solvent in combination with ultrasound probe, since the isolated components showed significantly lower selectivity for camphene and limonene, in addition to larger yield of polymeric by-products. These probably is consequence of slow changes induced to the catalyst by the catalytic reaction such as oxidation of SPION from magnetite to maghemite, decrease in the concentration and nature of the active acid sites, and change in the chemical environment of the Nb sites. The good performance and selectivity even after the fifth reuse cycle indicate the good stability of the catalyst and good efficiency of the magnetic recovery, associated with low leaching, demonstrating the high potentiality of the optimized material and process.

## Experimental section

### Materials

All chemicals were acquired commercially from Aldrich or Merck Company and used without further purification. Crude turpentine oil, pure α-pinene and β-pinene were donated by SOCER RB Company. Turpentine oil composition was evaluated by GC-MS using hexadecane as internal standard: 54–55% α-pinene, 44–45% β-pinene and 1–2% camphene. A Q700W Q-Sonica ultrasonic probe operating at frequency of 20 kHz was used in the experiments. The GC-MS analyzes were performed in a Shimadzu model GCMS-QP2010 ULTRA equipment. Niobium chloride (NbCl_5_) was kindly donated by Prof. Dr Thiago Canevari. Toluene, diethylene glycol, tetramethyl ammonium, hydrogen peroxide (30 wt%), tetrahydrofuran (THF) and ammonium hydroxide (99.8 wt%) were purchased from Merck®. Superparamagnetic iron oxide nanoparticles (SPIONs), 7 nm large, was prepared by thermo-decomposition process, as described previously.^35,57–59^

### Characterization methods

Transmission electron microscopy (TEM) images were obtained using a JEOL, model JEM 2100 FEG-TEM (at National Nanotechnology Laboratory – LNNANO, of the National Center for Energy and Materials Research– CNPEM, or at the Analytical Center of Institute of Chemistry of University of Sao Paulo) equipped with a LaB_6_ filament gun, Maximum acceleration voltage: 200 kV, Resolution: 0.23 nm (dot). The crystalline structures of samples were surveyed by X-ray diffractometry (XRD) using a Brucker D2 Phaser with Cu K_α_ radiation (*λ* = 1.5418 angstrom) at 20 keV. The Dispersive Energy X-ray fluorescence spectra (EDX) were obtained at 25 °C in a Shimadzu EDX-720 equipment, with a Rh tube as X-ray source, 15–50 kV voltage and a Si(Li) semiconductor detector cooled by liquid nitrogen. The solid samples were analyzed as powder placed onto Mylar® film in a 30 mm diameter sample holder, while liquid samples were placed onto Mylar® films in a 10 mm diameter sample holder and let dry under vacuum. The surface area and pore size were measured by nitrogen gas adsorption in a Gemini VII equipment, and isotherm curve analysis was carried out by BET method. The magnetization curves of solid samples were obtained using a vibrating sample magnetometer manufactured by EG&G Princeton Applied Research-model 4500. A QSONICA 700 watts ultrasonic probe was utilized in the reactions setting the pulse time to 15 s and the rest time to 15 s. The particles size and zeta potential measurements were carried out in a Malvern Zetasizer Nano ZS equipment using samples dispersed in water. The acidic sites were determined by pyridine adsorption assay as monitored by FTIR, and spectra acquired in a ALPHA Bruker Spectrophotometer, in transmission mode, with samples dispersed in KBr pellets. The chemical composition of the passive film was analyzed by X-ray photoelectron spectroscopy (XPS) using a ThermoVG K-alpha + spectrometer operating with Al-Kα radiation source. The pressure in the analysis chamber was 5 × 10^−8^ mPa and the spot size was 400 μm. The energy scale was calibrated with respect to the adventitious C 1s peak at 284.8 eV. Peak fitting was carried out with the Avantage v5.9912 software using a combination of Lorentzian and Gaussian line shapes and the Smart algorithm for background subtraction.

### Synthesis of SPION-Nb30 catalyst

The Nb precursor solution was prepared by dissolving 6.75 g of niobium(v) chloride (0.025 mols) in 200 mL of diethylene glycol (DEG) upon heating to approximately 180 °C for two hours. The light green solution was cooled to 50 °C and filtered using a quantitative paper filter to remove eventual impurities. SPIONs were prepared by thermo-decomposition reaction of iron(iii) precursor in a high boiling temperature solvent.^35,60^ Briefly, a solution containing 5 mmol of an iron(iii) complex was heated to 180 °C for 30 minutes, and the temperature increased to 230 °C for 30 minutes. After cooling, the product was precipitated out with tetrahydrofuran (THF) and the black solid isolated using a Nd_2_Fe_14_B magnet. Purification was carried out by successive cycles of redispersion of the solid in small volumes of water and precipitation with equal volume of THF. The surface of nanoparticles was activated by dispersing the solid in a 1 : 5 : 1 v/v mixture of NH_4_OH/H_2_O/H_2_O_2_ and keeping the suspension in an ultrasonic bath for 10 minutes. The solid was magnetically separated and washed copiously with deionized water and dried. Thus, 1.0 g of dried SPION were dispersed in 10 mL of tetramethylammonium hydroxide solution (TMAOH 0.1 mol L^−1^, pH ∼12), and added to 50 mL of 1.16 wt% solution of Nb precursor (0.936 g of Nb). The mixture was placed in the stainless steel (316) container of a high-pressure reactor, adjusting the temperature to 300 °C (inner part), under mechanical stirring (400 rpm). The reaction was performed at 300 °C and pressure of 46 psi, for 3 hours. After cooling, the solid was magnetically recovered, washed with deionized water, and dried at 120 °C.

## Isomerization reaction

The reaction was carried out by transferring 2 g of turpentine (14.7 mmol), 1 wt% of hexadecane, 2 mL of solvent (when applicable), 5 wt% of catalyst magnetite nanoparticles coated with niobium oxide (SPION-Nb30) and 5 wt% of phosphotungstic acid (HPW) into a glass flask. The reaction mixture was subjected to 30 minutes of sonication with a QSONICA ultrasound probe (700 W), setting the amplitude to 40 and the pulse sequence (15 seconds on followed by 15 seconds off). Every 10 minutes, samples were collected in a tube and mixed with a Na_2_CO_3_ solution (10 wt%) and 2 mL of ethyl acetate. After vortexing and settling stages, the organic layer was collected into a 2 mL vial and injected in a CG-MS equipment for analysis.

### Analysis by gas chromatography

All collected samples were analyzed by Gas Chromatography (GC), comparing the retention times of the compounds in the reaction mixture with those of standard compounds. The analyzes were performed in a Shimadzu model GCMS-QP2010 Ultra gas chromatography apparatus equipped with a Restek Rtx-5MS capillary column and a Flame Ionization Detector, using a high purity helium as carrier gas. The GC oven temperature was set at initial temperature of 50 °C, held for 5 minutes, increased at a rate of 4 °C min^−1^ up to 150 °C, and then increased to 240 °C at a rate of 15 °C min^−1^. The injector and detector temperatures were set to 240 °C. The concentrations of reactants and products were directly measured in the GC ChemStation system based on the area under each peak in the chromatograms (*A*_i_). The α-pinene conversion (*C*_α-pinene_), β-pinene conversion (*C*_β-pinene_) and *α*-terpineol selectivity (*S*_α-terpineol_) were calculated according to the following equations:
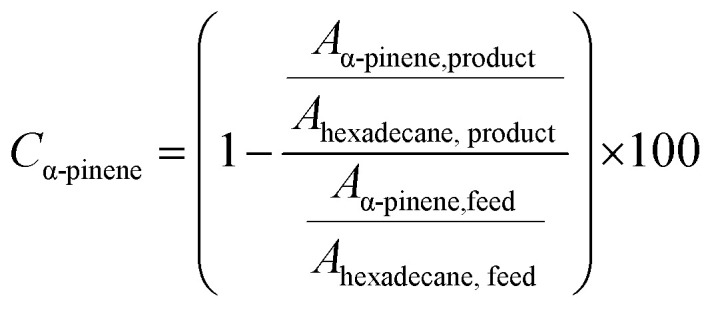

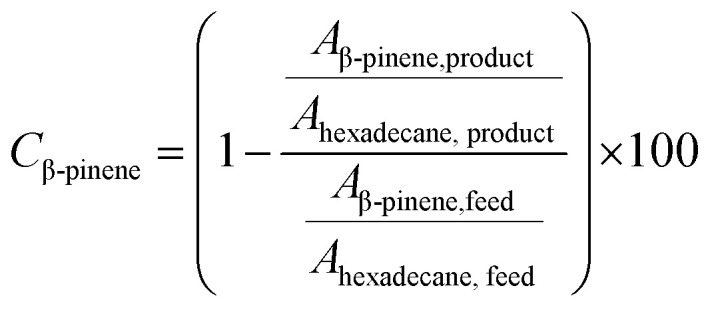

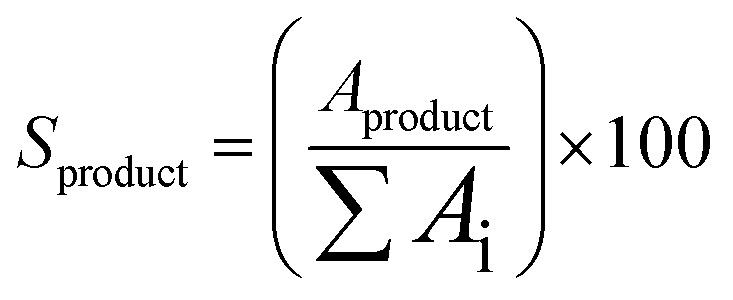


Representative chromatograms of the isomerization reaction products are available in the ESI.†

## Conflicts of interest

The authors declare no conflict of interest.

## Supplementary Material

RA-011-D1RA00012H-s001
