# Supplementary material for: Phosphotungstic acid impregnated niobium coated superparamagnetic iron oxide nanoparticles as recyclable catalyst for selective isomerization of terpenes
Source: RSC Adv. 2021 Apr 15;11(23):14203–12. doi: 10.1039/d1ra00012h (PMC8697717; doi:10.1039/d1ra00012h)
Supplement: RA-011-D1RA00012H-s001 [file RA-011-D1RA00012H-s001.pdf]

Electronic Supplementary Material (ESI) for RSC Advances.
This journal is © The Royal Society of Chemistry 2021

Supporting information for

Phosphotungstic Acid Impregnated Niobium Coated Superparamagnetic Iron Oxide Nanoparticles as Recyclable Catalyst for Selective Isomerization of Terpenes

Luccas Lossano Name,[a] Sergio Hiroshi Toma,[b] Helton Pereira Nogueira,[b] Luis Humberto Avanzi,[c] Rafael dos Santos Pereira,[d] Luis Fernando Peffi Ferreira,[a] Koiti Araki,[b] Rodrigo Cella *[a], and Marcos Makoto Toyama*[b]

[a.] *Msc. L. L. Name, Dr. L. F. P. Ferreira, Dr. R. Cella (r.cella@fei.edu.br)*
Department of Chemistry Engineering
FEI University
Address Av. Humberto de Alencar Castelo Branco, 3972B- Assunção – São Bernardo do Campo – São Paulo – Brasil – CEP 09850-901
[b.] *Dr. S. H. Toma, Dr. Helton P. Nogueira, Dr. K. Araki, Dr. M. M. Toyama (marcosmakotoyama@gmail.com)*
Department of Fundamental Chemistry
Chemistry Institute – São Paulo University - IQUSP
Address 2: Av. Professor Lineu Prestes, 748 – CEP 05508-000 - Cidade Universitária – São Paulo – SP – Brasil
[c.] *Dr. L. H. Avanzi*
Department of Physics
FEI University
Address 3: Av. Humberto de Alencar Castelo Branco, 3972B - Assunção – São Bernardo do Campo – São Paulo – Brasil – CEP 09850-901
[d.] *Msc R. S. Pereira Department of Physics*
Universidade Federal do ABC, Centro de Ciências Naturais e Humanas
Address 3: Avenida dos Estados, 5001 - Bloco A - Torre 3 - Lab. L704-3 -09210580 – Bangu- Santo André, SP - Brasil

1. REPRESENTATIVE GC CHROMATOGRAMS

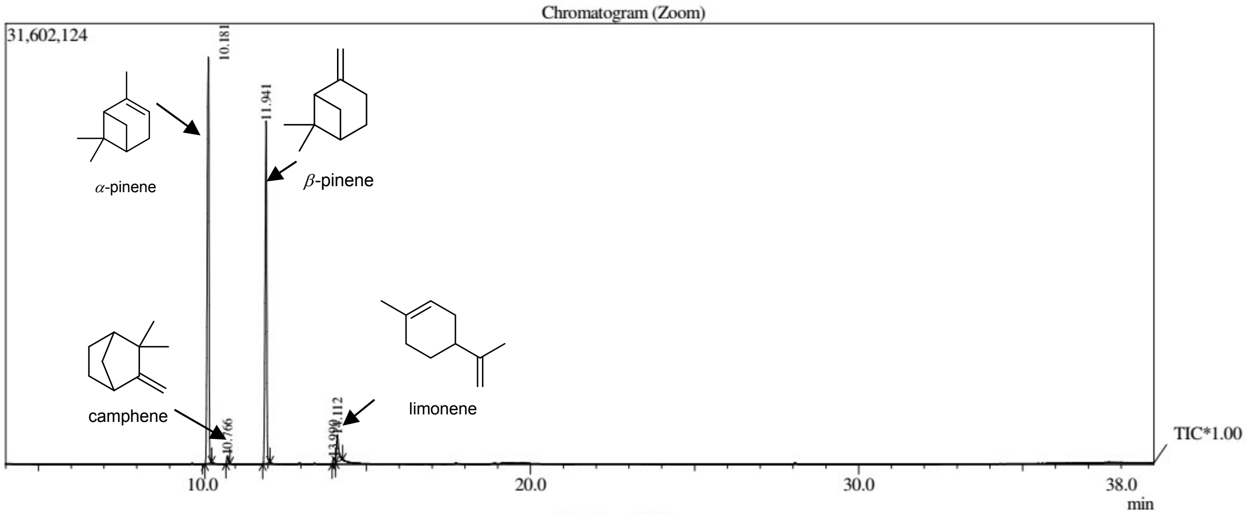

Peak Report TIC

Peak#	R.Time	I.Time	F.Time	Area	Area%	Height	Height%	A/H	Mark	Name
1	10.181	10.075	10.285	106243095	52.17	29112401	51.55	3.65		
2	10.766	10.715	10.835	1752516	0.86	534154	0.95	3.28		
3	11.941	11.840	12.060	84529169	41.51	24524352	43.42	3.45		
4	13.990	13.950	14.060	1969346	0.97	377418	0.67	5.22		
5	14.112	14.060	14.270	9156766	4.50	1927892	3.41	4.75	V	
				203650892	100.00	56476217	100.00			

Spectrum

Figure S1: Typical turpentine oil chromatogram (it can have variation).

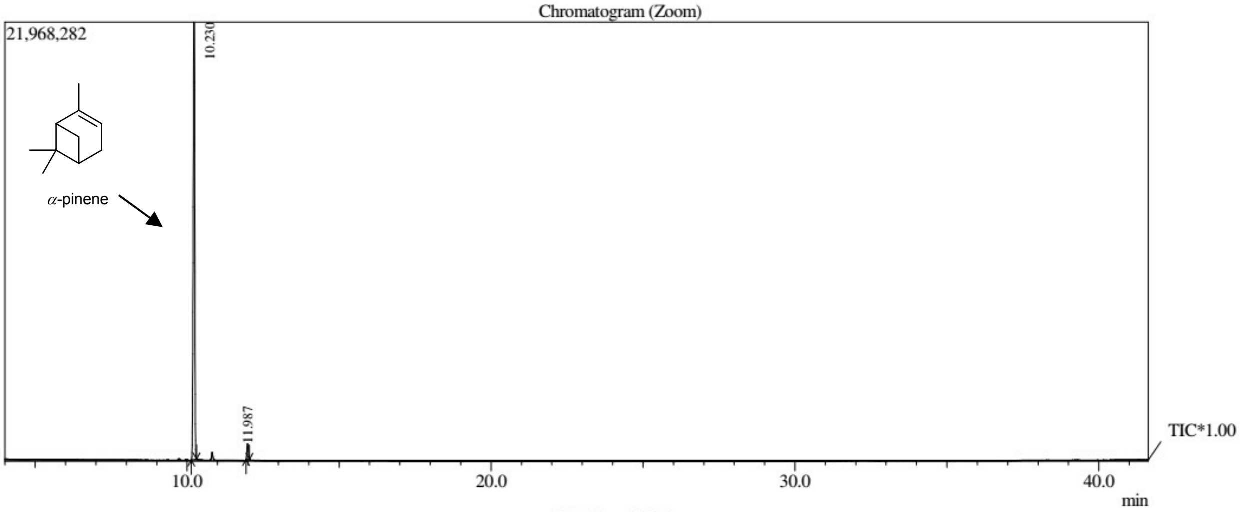

Peak Report TIC

Peak#	R.Time	I.Time	F.Time	Area	Area%	Height	Height%	A/H	Mark	Name
1	10.230	10.140	10.305	84067835	97.21	26690901	97.14	3.15		
2	11.987	11.940	12.050	2415070	2.79	784673	2.86	3.08		
				86482905	100.00	27475574	100.00			

Figure S2: Chromatogram of standard α-pinene.

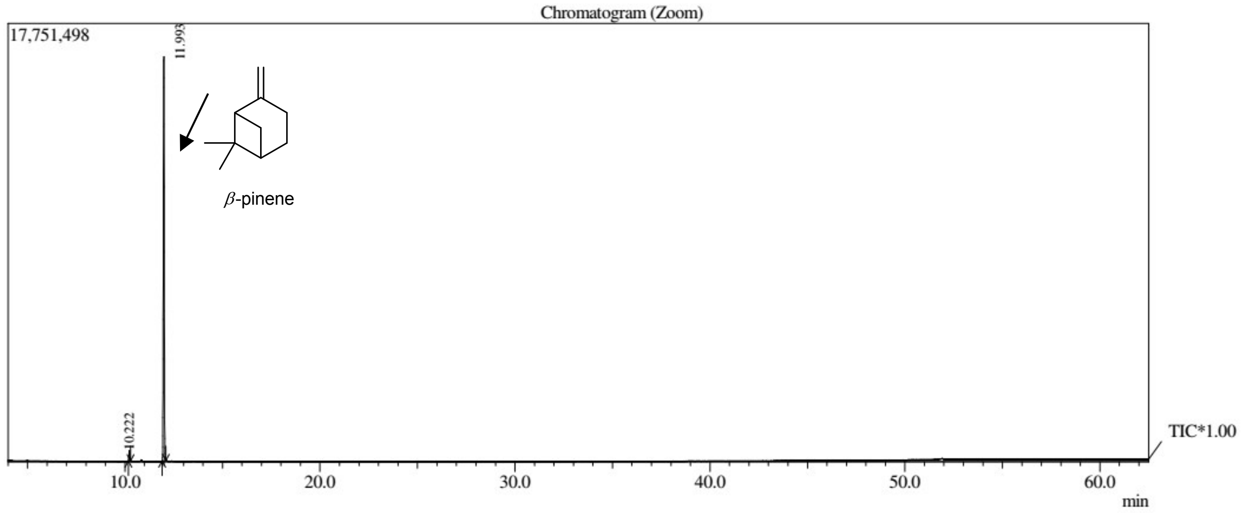

Peak Report TIC

Peak#	R.Time	I.Time	F.Time	Area	Area%	Height	Height%	A/H	Mark	Name
1	10.222	10.170	10.290	1281494	2.44	422573	2.52	3.03		
2	11.993	11.905	12.090	51294618	97.56	16316420	97.48	3.14		
				52576112	100.00	16738993	100.00			

Figure S3: Chromatogram of standard *β*-pinene.

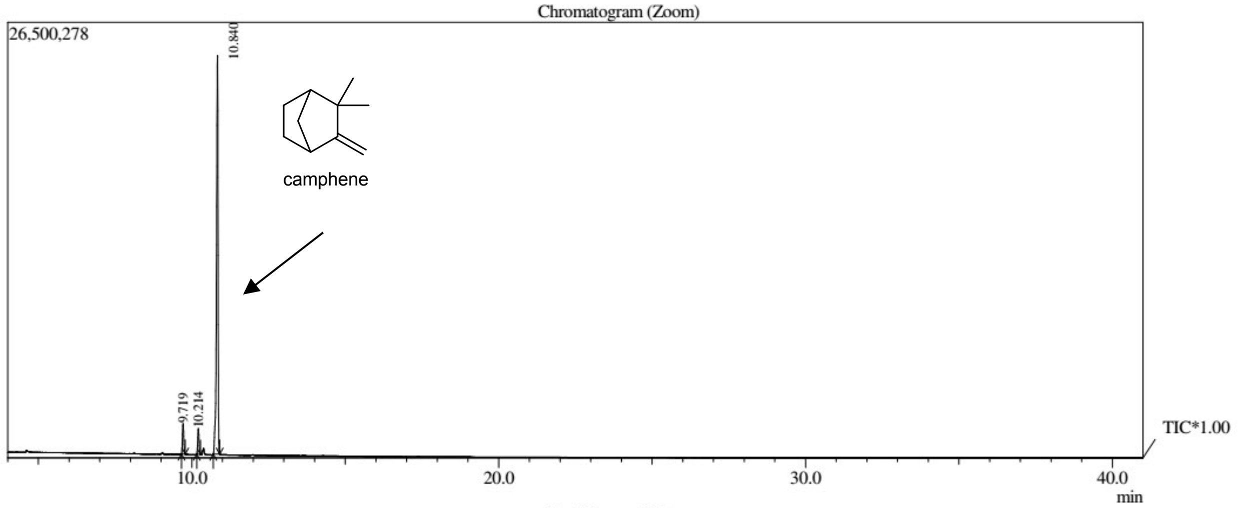

Peak Report TIC

Peak#	R.Time	I.Time	F.Time	Area	Area%	Height	Height%	A/H	Mark	Name
1	9.719	9.665	9.785	5588544	5.93	1854843	6.70	3.01		
2	10.214	10.160	10.280	4713198	5.00	1557643	5.63	3.03		
3	10.840	10.705	10.915	83934688	89.07	24258360	87.67	3.46		
				94236430	100.00	27670846	100.00			

Figure S4: Chromatogram of standard camphene.

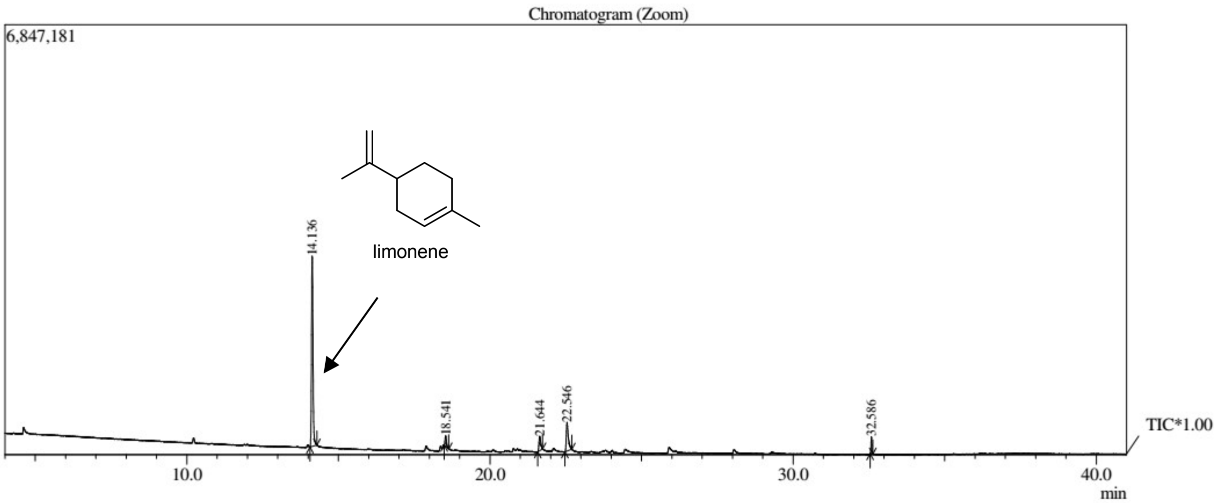

Peak Report TIC

Peak#	R.Time	I.Time	F.Time	Area	Area%	Height	Height%	A/H	Mark	Name
1	14.136	14.060	14.290	10184051	71.11	3019249	72.83	3.37		
2	18.541	18.490	18.640	554899	3.87	170048	4.10	3.26		
3	21.644	21.580	21.740	935367	6.53	227775	5.49	4.11		
4	22.546	22.475	22.700	2149212	15.01	456203	11.01	4.71		
5	32.586	32.545	32.635	498747	3.48	272113	6.56	1.83		
				14322276	100.00	4145388	100.00			

Figure S5: Chromatogram of standard limonene.

C:\GCsolution\Data\Cella\Makoto\RCII-72B-T0.qgd

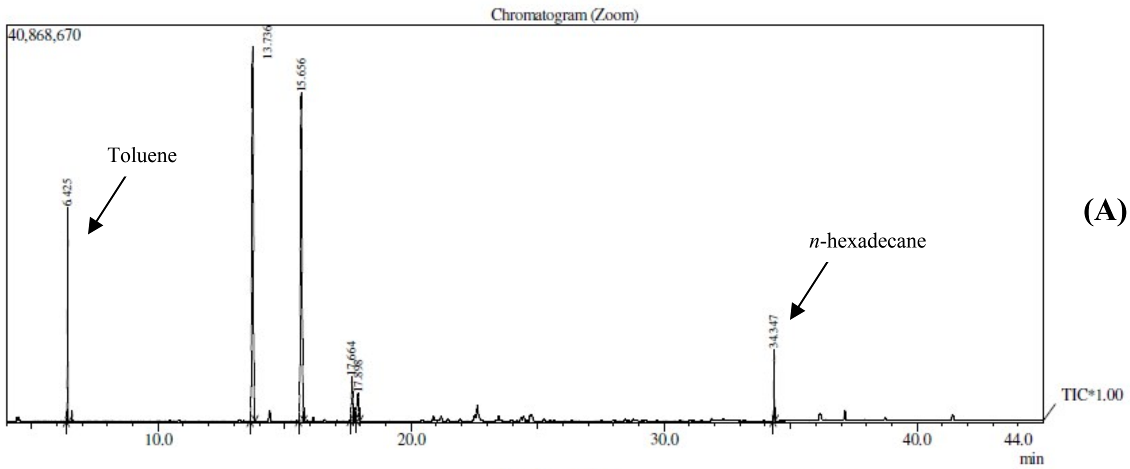

(A)

Peak Report TIC

Peak#	R.Time	I.Time	F.Time	Area	Area%	Height	Height%	A/H	Mark	Name
1	6.425	6.365	6.445	30110969	7.76	22132769	20.25	1.36		
2	13.736	13.630	13.830	169420856	43.69	38640380	35.36	4.38		
3	15.656	15.555	15.755	146330400	37.73	33857767	30.98	4.32		
4	17.664	17.595	17.740	15839283	4.08	4519275	4.14	3.50		
5	17.898	17.805	17.975	13405882	3.46	2778970	2.54	4.82		
6	34.347	34.305	34.395	12694316	3.27	7344709	6.72	1.73		
				387801706	100.00	109273870	100.00			

Spectrum

C:\GCsolution\Data\Cella\Makoto\RCII-72B-1R.qgd

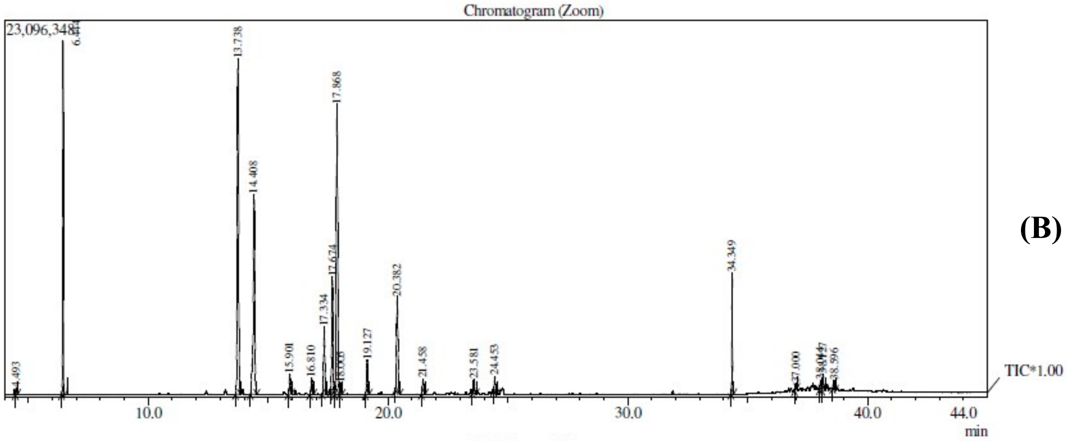

(B)

Peak Report TIC

Peak#	R.Time	I.Time	F.Time	Area	Area%	Height	Height%	A/H	Mark	Name
1	4.493	4.450	4.550	1116809	0.31	411541	0.38	2.71	V	
2	6.444	6.375	6.465	32965713	9.17	21951271	20.26	1.50		
3	13.738	13.635	13.845	84382055	23.48	20700299	19.10	4.08		
4	14.408	14.250	14.520	55929202	15.57	12350200	11.40	4.53		
5	15.901	15.820	15.985	4504448	1.25	1223780	1.13	3.68		
6	16.810	16.735	16.900	3898420	1.08	1027030	0.95	3.80		
7	17.334	17.245	17.430	14971231	4.17	4170132	3.85	3.59		
8	17.674	17.595	17.765	26394825	7.35	7162902	6.61	3.68		
9	17.868	17.765	17.960	71099234	19.79	17898584	16.52	3.97		
10	18.005	17.960	18.090	2636353	0.73	683221	0.63	3.86	V	
11	19.127	19.045	19.215	7706377	2.14	2146512	1.98	3.59		
12	20.382	20.290	20.500	23146361	6.44	5886549	5.43	3.93		
13	21.458	21.385	21.545	3539185	0.99	937706	0.87	3.77		
14	23.581	23.515	23.675	3115018	0.87	852712	0.79	3.65		
15	24.453	24.395	24.535	3241373	0.90	973719	0.90	3.33		
16	34.349	34.295	34.410	12616617	3.51	7446935	6.87	1.69		
17	37.000	36.955	37.050	908340	0.25	389396	0.36	2.33		
18	38.044	37.985	38.085	1804536	0.50	601800	0.56	3.00		
19	38.127	38.085	38.230	3563666	0.99	996846	0.92	3.57	V	
20	38.596	38.535	38.665	1764771	0.49	556042	0.51	3.17		
				359304534	100.00	108367177	100.00			

Spectrum

Figure S6: Chromatogram of isomerization reaction under HIU, conditions described on Run 1, Table 4. A) Time zero B) 30 minutes.

C:\GCsolution\Data\Cella\Makoto\RCII-72B-2R.qgd

Chromatogram (Zoom)

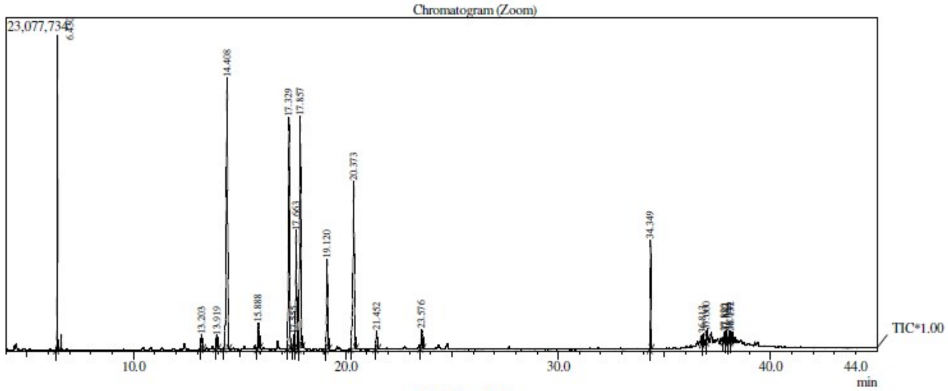

(A)

Peak Report TIC

Peak#	R.Time	I.Time	F.Time	Area	Area%	Height	Height%	A/H	Mark	Name
1	6.430	6.365	6.450	31290107	8.08	21935643	18.63	1.43		
2	13.203	13.145	13.275	3658327	0.94	994221	0.84	3.68		
3	13.919	13.865	13.990	3172685	0.82	901679	0.77	3.52		
4	14.408	14.260	14.505	79031951	20.40	18831381	16.00	4.20		
5	15.888	15.825	15.965	6178136	1.59	1763871	1.50	3.50		
6	17.329	17.240	17.420	59551799	15.37	16033865	13.62	3.71		
7	17.555	17.500	17.595	3259889	0.84	950451	0.81	3.43		
8	17.663	17.595	17.750	30606583	7.90	8218895	6.98	3.72	V	
9	17.857	17.770	17.955	61811150	15.95	16108875	13.68	3.84		
10	19.120	19.040	19.205	22904019	5.91	6231748	5.29	3.68		
11	20.373	20.230	20.475	52336615	13.51	11615020	9.87	4.51		
12	21.452	21.390	21.530	4609732	1.19	1252758	1.06	3.68		
13	23.576	23.515	23.655	4620645	1.19	1270750	1.08	3.64		
14	34.349	34.305	34.395	12874461	3.32	7524250	6.39	1.71		
15	36.813	36.765	36.870	1944229	0.50	673644	0.57	2.89		
16	37.000	36.960	37.040	1411769	0.36	645585	0.55	2.19		
17	37.820	37.780	37.850	1496378	0.39	639392	0.54	2.34		
18	37.882	37.850	37.935	1632492	0.42	680147	0.58	2.40	V	
19	38.044	38.000	38.085	1936953	0.50	704660	0.60	2.75		
20	38.132	38.085	38.230	3125325	0.81	740757	0.63	4.22	V	
				387453245	100.00	117717592	100.00			

Spectrum

C:\GCsolution\Data\Cella\Makoto\RCII-72B-3R.qgd

Chromatogram (Zoom)

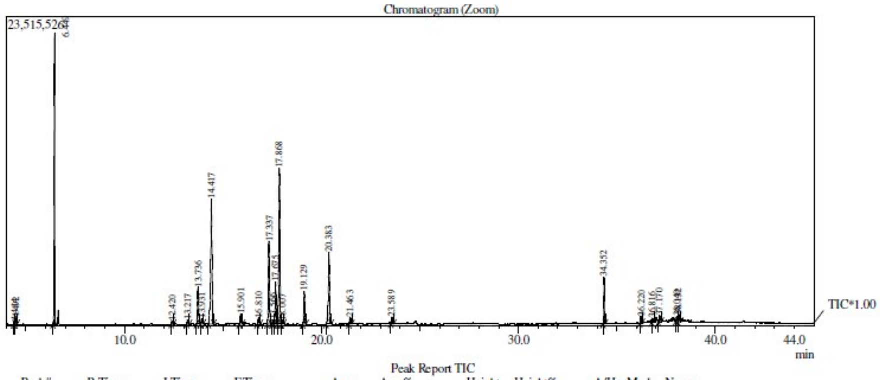

(B)

Peak Report TIC

Peak#	R.Time	I.Time	F.Time	Area	Area%	Height	Height%	A/H	Mark	Name
1	4.414	4.370	4.450	707336	0.31	270888	0.37	2.61		
2	4.492	4.450	4.555	1171295	0.51	427943	0.58	2.74	V	
3	6.449	6.370	6.470	36071882	15.71	22355567	30.19	1.61		
4	12.420	12.370	12.495	710403	0.31	193221	0.26	3.68		
5	13.217	13.160	13.295	1010960	0.44	274099	0.37	3.69		
6	13.736	13.650	13.835	10696138	4.66	2801971	3.78	3.82		
7	13.931	13.850	14.005	1404765	0.61	377925	0.51	3.72		
8	14.417	14.255	14.530	42010902	18.29	9543518	12.89	4.40		
9	15.901	15.825	15.995	2687533	1.17	742607	1.00	3.62		
10	16.810	16.730	16.895	1797803	0.78	461568	0.62	3.89		
11	17.337	17.235	17.440	23589512	10.27	6317544	8.53	3.73		
12	17.566	17.500	17.605	1041048	0.45	288897	0.39	3.60		
13	17.675	17.605	17.765	11722967	5.10	3209694	4.33	3.65	V	
14	17.868	17.765	17.960	45678942	19.89	11857692	16.01	3.85	V	
15	18.007	17.960	18.090	1076874	0.47	273664	0.37	3.94	V	
16	19.129	19.040	19.225	8836472	3.85	2487736	3.36	3.55		
17	20.383	20.230	20.505	22843257	9.95	5478365	7.40	4.17		
18	21.463	21.390	21.550	1995909	0.87	524327	0.71	3.81		
19	23.589	23.525	23.680	1889809	0.82	504496	0.68	3.75		
20	34.352	34.295	34.410	6085137	2.65	3546799	4.79	1.72		
21	36.220	36.160	36.285	1269737	0.55	527358	0.71	2.41		
22	36.816	36.760	36.885	745145	0.32	234952	0.32	3.17		
23	37.170	37.110	37.265	1491782	0.65	460926	0.62	3.24		
24	38.049	37.980	38.090	1220438	0.53	395127	0.53	3.09		
25	38.132	38.090	38.235	1889168	0.82	485234	0.66	3.89	V	
				229645214	100.00	74042118	100.00			

Spectrum

C:\GCsolution\Data\Cella\Makoto\RCII-72B-4R.QGD

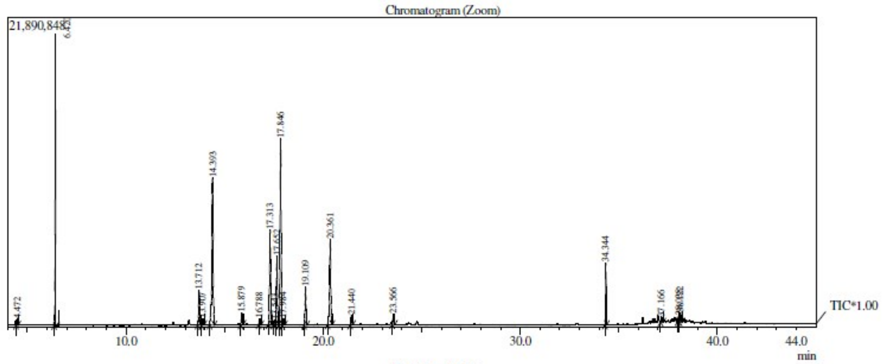

(C)

Peak Report TIC

Peak#	R.Time	I.Time	F.Time	Area	Area%	Height	Height%	A/H	Mark	Name
1	4.472	4.430	4.530	1138875	0.47	424027	0.55	2.69	V	
2	6.420	6.350	6.440	30221303	12.43	20816084	26.83	1.45		
3	13.712	13.630	13.800	9561883	3.93	2477788	3.19	3.86		
4	13.907	13.825	13.975	1603102	0.66	421671	0.54	3.80		
5	14.393	14.240	14.495	46164954	18.99	10479301	13.50	4.41		
6	15.879	15.815	15.955	3036028	1.25	863163	1.11	3.52		
7	16.788	16.735	16.860	1356824	0.56	393338	0.51	3.45		
8	17.313	17.225	17.405	25160284	10.35	6741324	8.69	3.73		
9	17.543	17.490	17.580	1244895	0.51	367118	0.47	3.39		
10	17.652	17.580	17.740	18004625	7.41	4891059	6.30	3.68	V	
11	17.846	17.750	17.935	50869434	20.92	13271222	17.10	3.83		
12	17.984	17.935	18.060	1745083	0.72	461233	0.59	3.78	V	
13	19.109	19.030	19.190	9741303	4.01	2717210	3.50	3.59		
14	20.361	20.225	20.470	25601462	10.53	6078000	7.83	4.21		
15	21.440	21.375	21.520	2617214	1.08	706644	0.91	3.70		
16	23.566	23.505	23.645	2567099	1.06	703417	0.91	3.65		
17	34.344	34.295	34.395	7530254	3.10	4384575	5.65	1.72		
18	37.166	37.120	37.255	1454039	0.60	363191	0.47	4.00		
19	38.038	37.990	38.080	1197210	0.49	401856	0.52	2.98		
20	38.122	38.080	38.220	2319964	0.95	635737	0.82	3.65	V	
				243135835	100.00	77597958	100.00			

Spectrum

Figure S7: Chromatograms of isomerization reaction with recycled catalyst, under HIU, conditions described on Runs 2-4, Table 4. A) 2nd reclycling B) 3rd reclycling C) 4th reclycling.

2. X-RAY PHOTOELECTRON SPECTROSCOPY (XPS)

The figures below correspond to the high-resolution spectra of phosphorus (P 2p) and carbon (C 1s) and the survey spectra of pure SPION, SPION modified as SPIO-Nb30 and SPION-Nb30 + HPW in run 1 and Run 5, showed in Figure **S8** e **S9**, respectively.

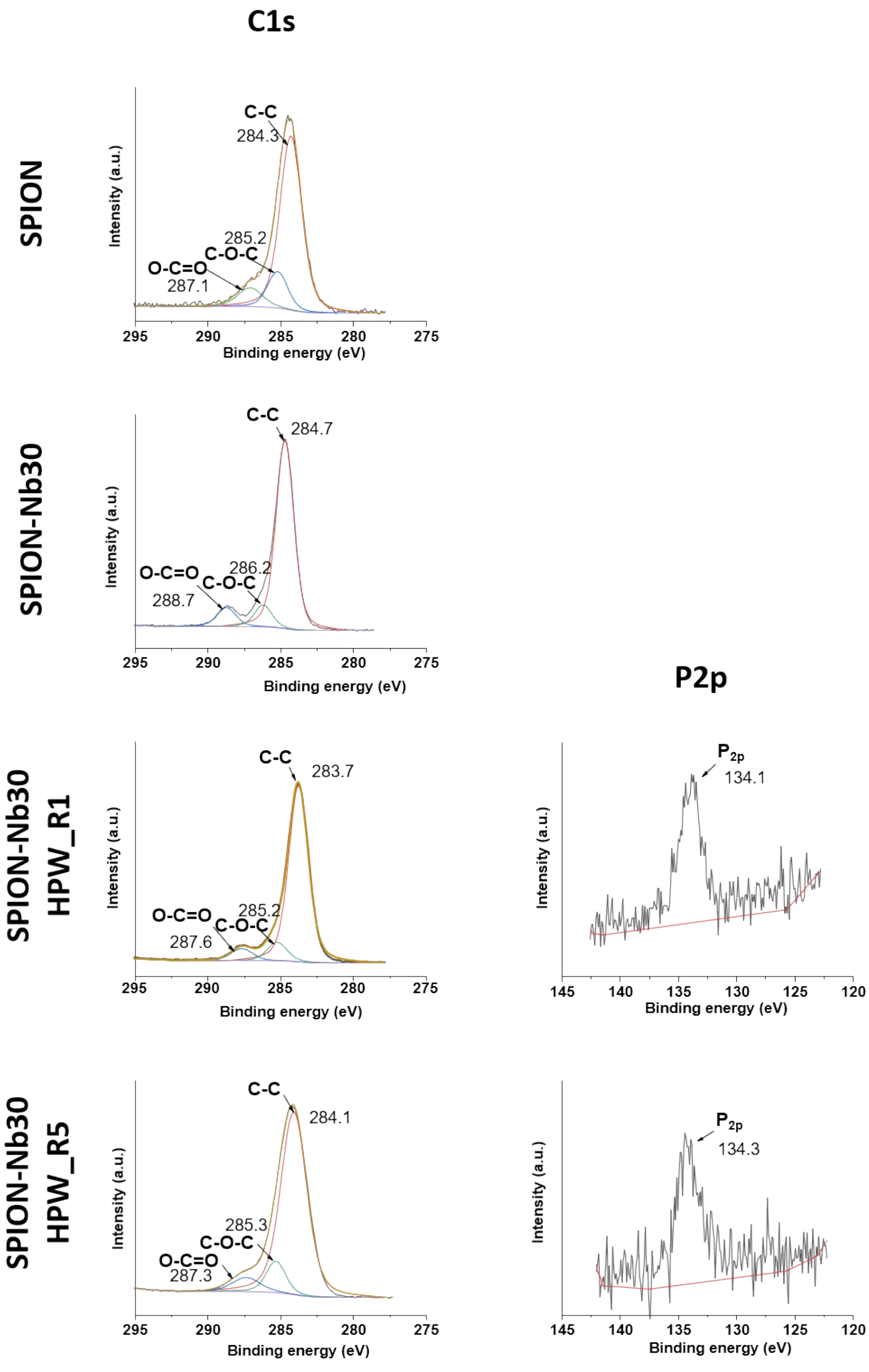

Figure S8: High resolution XPS spectra of SPION, SPION-NB30, SPION-Nb30@HPW-R1 (run 1), and SPION-Nb30@HPW-R5 (Run 5) magnetically recovered from the reaction mixture after the first and fifth catalytic cycle.

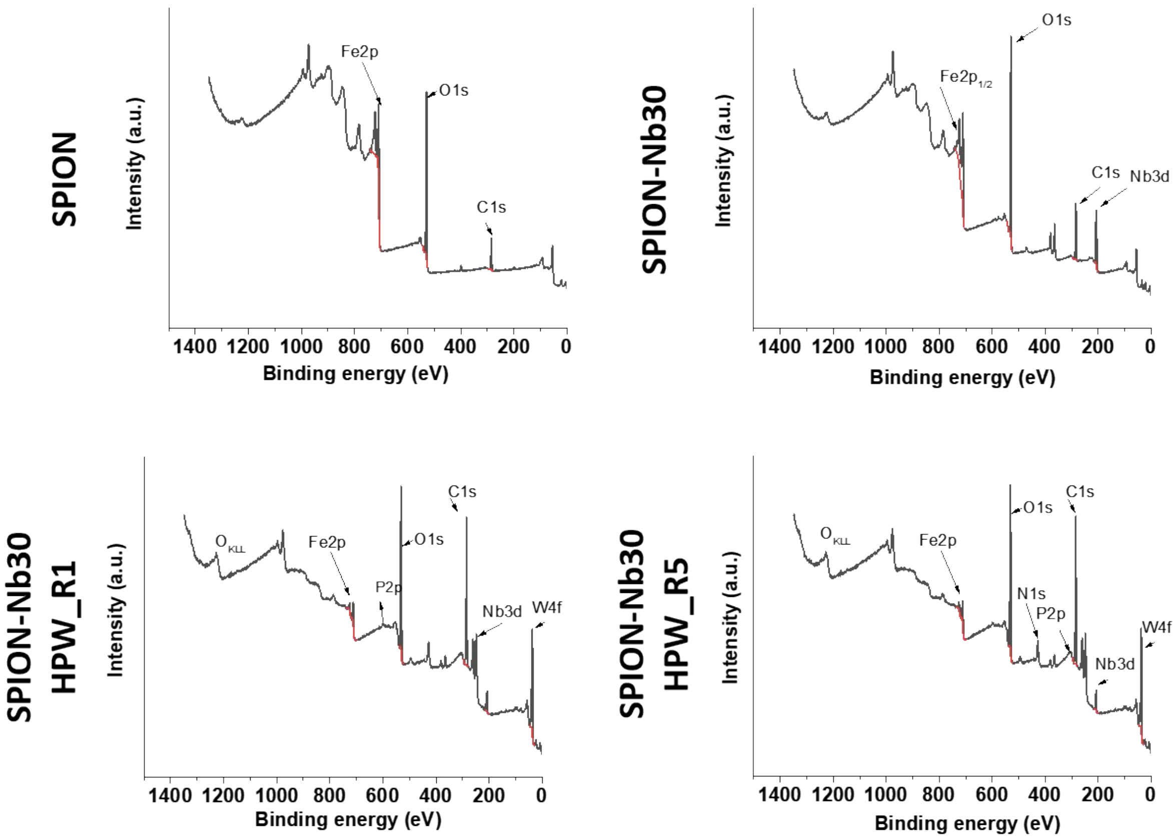

Figure S9: Survey XPS spectra (Survey) of SPION, SPION-NB30, SPION-Nb30@HPW-R1 (run 1), and SPION-Nb30@HPW-R5 (Run 5) magnetically recovered from the reaction mixture after the first and fifth catalytic cycle.
